# Opsoclonus-Myoclonus With Anti-YO Antibodies Revealing Breast Cancer: A Case Report

**DOI:** 10.7759/cureus.60452

**Published:** 2024-05-16

**Authors:** Yousfi Samah, Boulehoual Sahar, Yassine Mebrouk

**Affiliations:** 1 Department of Neurology, Mohamed VI University Hospital, Mohamed First University, Faculty of Medicine, Oujda, MAR

**Keywords:** cerebral ataxia, paraneoplastic neurological syndromes, anti-yo antibodies, breast cancer, opsoclonus-myoclonus

## Abstract

Opsoclonus myoclonus syndrome (OMS) is a rare neurological disorder characterized by irregular, continuous, and chaotic eye saccades accompanied by myoclonus, defined by brief, shock-like muscle spasms in the arms or legs. This syndrome often presents with additional features such as cerebellar syndrome, nycthemeral rhythm disorders, hallucinosis, and irritability-type behavioral disorders. In adults, OMS is predominantly paraneoplastic, necessitating screening for onconeural antibodies (ONA). While specific medications for OMS are lacking, addressing the underlying cause may ameliorate its clinical manifestations. The presence of opsoclonus-myoclonus should prompt urgent and thorough investigation for an underlying cancer, given its frequent association with paraneoplastic neurological syndrome (PNS). Here, we present the case of a 39-year-old patient with opsoclonus associated with cerebellar ataxia, revealing a breast neoplasm with positive anti-YO antibodies. Through the review of the literature, we discuss the epidemiological, clinical, diagnostic, and therapeutic aspects of this rare situation.

## Introduction

Opsoclonus myoclonus syndrome (OMS), also known as Dancing Eyes-Dancing Feet syndrome and Kinsbourne syndrome, is one of the paraneoplastic neurological syndrome (PNS) clinical presentations. It is extremely rare, impacting as few as 1 in 10,000,000 individuals annually. It is observed in 2% to 3% of children [1].

The exact immunopathogenesis of OMS remains uncertain. However, there is growing acknowledgment that both humoral and cell-mediated immune mechanisms are involved. Although changes in the synaptic weighting of saccadic burst neuron circuits in the brainstem may produce saccadic oscillations, clinical correlation is lacking [2].

The recognition of this should trigger an investigation for onconeural antibodies (ONA) in either the serum or cerebrospinal fluid, facilitating the diagnosis of the associated cancer. The neurological prognosis is further contingent upon the promptness of this diagnosis and the initiation of cancer treatment [3].

## Case presentation

A 39-year-old woman with no medical history presented to the hospital with progressive neurological deterioration, including binocular diplopia with an unsteady gait that evolved for three months. The admission neurological examination revealed an opsoclonus-myoclonus with disordered eye movements with multidirectional oscillations, impairment of the sixth cranial nerve associated with palatal and facial myocloni, and a state-kinetic cerebellar syndrome.

The breast examination revealed an enlarged left breast with a painless multi-lobulated nodule measuring 6 cm in the upper outer quadrant of the left breast, located in the retro-mammary region. A left axillary lymphadenopathy was palpable, and a milky discharge from the nipple was present. No skin changes were noted. She was subsequently referred for a breast ultrasound and a mammogram.

A gadolinium-enhanced brain and spine MRI was normal. The levels of vitamins E and B12, as well as thyroid hormones, were normal. Initial metabolic and infectious investigations were normal. Serum was analyzed for the presence of onco-neural auto-antibodies; it was positive for anti-Yo antibodies and negative for AC anti-Hu, AC anti-RI, AC anti-amphiphysin, AC anti-Ma2, AC anti-AMPAR (anti-α-amino-3-hydroxy-5-methyl-4-isoxazolepropionic acid receptor), and AC anti-Tr.

Mammography showed a dense mass in the upper outer quadrant of the left breast, measuring approximately 5 cm in its largest dimension, with irregular contours and associated skin thickening and the presence of multiple supra-centimetric left axillary adenomegalies. There was no evidence of calcification. Sono-mammography of the opposite breast and axilla was normal.

**Figure 1:**
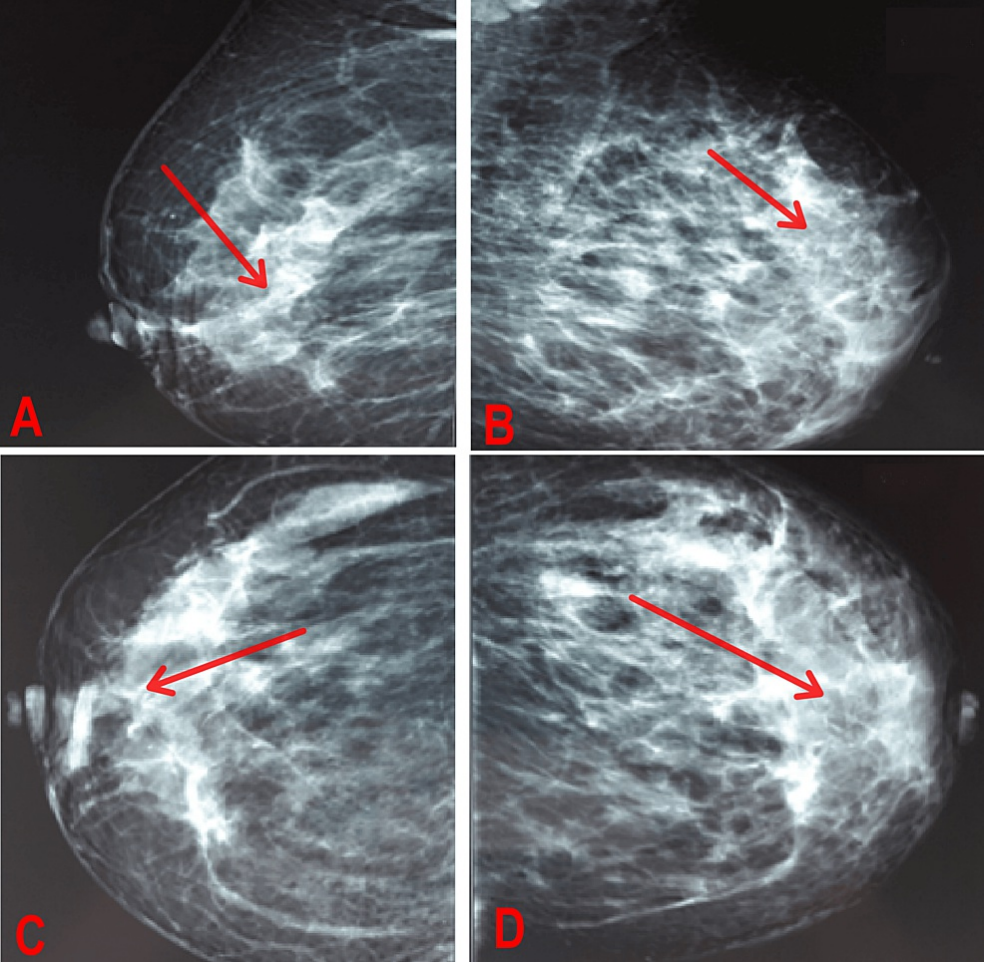
Mammography of the left and right breast showing a mass (arrow) in the upper outer quadrant of the left breast, measuring 5 cm, with irregular contours, classified as BIRADS V (A) Right mediolaterl view; (B) left mediolateral view; (C) right craniocaudal view; (D) left craniocaudal view

The biopsy of the breast mass yielded an infiltrating poorly differentiated mammary carcinoma grade II SBR, an intermediate nuclear-grade intra-canalicular component with vascular emboli. The diagnosis of a paraneoplastic neurological syndrome characterized by opsoclonus-myoclonus and cerebellar ataxia related to breast neoplasms has been established.

Comprehensive neoplastic screening (gastroscopy, PET/CT) revealed no evidence of malignancy, and an autoimmune screen was negative. The tumor was classified as T4bN2M0. The patient underwent neoadjuvant chemotherapy, followed by a mastectomy with lymph node dissection. The patient's neurological condition remained stable after a 12-month follow-up.

## Discussion

Opsoclonus-myoclonus-ataxia syndrome (OMAS) is probably a neuroimmunological disease, frequently indicative of a paraneoplastic process. However, it has also been reported for infectious, autoimmune, toxic, or metabolic causes. In adults, paraneoplastic etiologies are the most common (39% of cases). It most often precedes the diagnosis of a tumor, predominantly represented by small-cell lung cancers and breast adenocarcinomas [3]. However, cases have also been reported in association with ovarian teratomas, non-small cell lung cancers, non-Hodgkin lymphomas, and head and neck cancers, as well as thyroid cancers or even melanomas [4].

The pathophysiological mechanism of opsoclonus is not yet elucidated. Many theories have been proposed. The main hypothesis is that there is a dysfunction, mediated by an immunological mechanism, of structures in the brainstem and cerebellum, including the cerebellar fastigial nucleus and the omnipause neurons located in the nucleus of the interpositus raphe. The exact mechanism by which these structures are damaged is uncertain, but many arguments support an autoimmune, particularly humoral, involvement. The humoral immunopathogenesis in paraneoplastic opsoclonus has been elucidated by the detection of a multitude of antineuronal antibodies: Ri, YO, Hu, Ma1, Ma2, amphiphysin, CV2, Zic2, and neurofilaments. The autoantigens would be located at the synapse or on the cell surface, and the antibodies would have a transient effect on nerve cells [5,6].

Every patient presenting with OMS requires an extensive diagnostic investigation consisting of morphological examinations (especially cerebral MRI), which generally do not reveal any abnormalities. A thoracoabdominopelvic CT scan and, in women, mammography coupled with a thorough gynecological examination should be performed. If inconclusive, a PET scan should ideally be conducted for all patients. In instances where the initial investigation yields negative results, a follow-up assessment and reinvestigation to rule out hidden tumors should be conducted a few months after the initial workup [7].

Over the past 20 years, the identification of onconeural antibodies has marked a significant breakthrough, supporting the hypothesis of an autoimmune mechanism. Their presence suggests the paraneoplastic nature of the neurological presentation and guides the search for cancer based on the type of antibodies identified. OMS is associated with several antibodies: anti-Hu, anti-YO (PCA-1), anti-Tr, anti-Ri, and anti-Ma2 [8-10].

Anti-YO antibodies are associated with certain gynecological cancers (ovarian and, rarely, uterine), breast cancer, lung cancer, and gastric cancer. Anti-YO antibodies are part of the first class of antibodies targeting intracellular antigens. A neuronal protein expressed in the cytoplasm of Purkinje cells in the cerebellum, known as CDR2, has been identified. This protein is also expressed in breast and ovarian tumors. The discovery of these antibodies should prompt a search for this type of tumor [11].

In our patient, examinations conducted following the discovery of a high level of anti-YO antibodies revealed breast cancer. In the case of a negative etiological investigation, an exploratory laparotomy is recommended to screen for sub-radiological ovarian cancer [12].

The neurological functional prognosis of PNS associated with this type of AON is poor. Despite the immunological mechanisms underlying most paraneoplastic syndromes, the outcomes of immunotherapy, plasma exchanges, immunoadsorption, and corticosteroids have been disappointing. In this case, the treatment for opsoclonus-myoclonus is that of the underlying tumor [3].

## Conclusions

The identification of an OMS should incite paraclinical investigations to enable the diagnosis of the underlying cancer. These include a CT scan, MRI imaging of the neck, chest, abdomen, and pelvis, mammography in women, and PET-CT in case of a negative work-up. Directed or broad screening for paraneoplastic antibodies should be considered, although the absence of detectable antibodies does not always rule out a paraneoplastic syndrome. The neurological prognosis also depends on the early diagnosis and treatment of the cancer.
